# Therapeutic Effects of Adrenocorticotropic Hormone ACTH in Children with Severely Intractable Seizure

**Published:** 2017

**Authors:** Jafar NASIRI, Azam SARAJAN, Mehri SALARI, Maryam SEDGHI

**Affiliations:** 1 Departement of Pediatric Neurology and Child Growth and Development Research Center, Isfahan University of Medical Sciences, Isfahan, Iran

**Keywords:** ACTH, Children, Epilepsy, Seizures, Intractable

## Abstract

**Objective:**

Treatment of intractable seizures other than spasms is difficult and controversial.

There are few studies on efficacy of adrenocorticotropic hormone (ACTH) in treatment of patients with intractable seizure.

**Materials & Methods:**

Twenty-five patients with intractable seizure other than spasm including 14 boys and 11 girls with median age of 58 months referred to university clinics of Pediatric Neurology in Isfahan, Iran, during 2014-2015 were prospectively investigated. ACTH was administrated according to our protocol. All cases were followed regularly and assessed for response to treatment and probable side effects, 3 wk after beginning of ACTH therapy and three months after the ACTH therapy. EEG finding were recorded before and three months after the end of ACTH therapy. Statistical analysis using Freidman test and Wilcoxon signed – rank test were performed in order to compare seizure frequency and EEG changes, respectively.

**Results:**

Mean A significant reduction (>80%) in seizure frequency in 11 cases (44%) and moderate reduction (50%-80%) in 7 (28%) after 3 wk of ACTH therapy.

Despite initial positive response, recurrence of seizure was observed in 7 out of 18 cases with favorable initial response within 3 months after ACTH therapy cessation. The comparison of EEG finding before and 3 months after ACTH therapy using Wilcoxon signed – rank test showed significant differences.

**Conclusion:**

ACTH therapy may be useful in treatment of children with intractable seizures who are resistant to usual antiepileptic drugs. However further studies should be performed to determine the long-term efficacy of ACTH in treatment of intractable seizure.

## Introduction

Epilepsy and epileptic syndromes are a heterogeneous group of conditions with a prevalence of 5.3- 8.8 per 1000 in children below 13 yr of age (1, 2). Overall, 20%-25% of epileptic patients are resistant to all classical antiepileptic drugs. An intractable seizure is defined by therapeutic failure of seizure freedom in response to two tolerated and appropriately chosen antiepileptic drugs at reasonable doses. 

Frequency of seizures and duration of non-controlled epilepsy was considered as criteria for intractable seizure (1, 3).

Although corticosteroids and adrenocorticotropic hormone (ACTH) as a treatment option are used in different epilepsies and epileptic syndromes, the efficacy of ACTH and steroids in controlling intractable seizure other than spasms has been studied only in few studies (4-8). ACTH therapy is also effective for epilepsies other than infantile spasm (6, 9-13). 

In this article, we report our experience using ACTH in the treatment of 25 patients with generalized seizures other than spasms who had been refractory to all usual AEDs.

## Materials & Methods

Twenty-five patients with intractable seizure other than spasm including 14 boys and 11 girls with median age of 58 months referred to university clinics of pediatric neurology in Isfahan, Iran, during 2014-2015 were prospectively investigated in this study. 

The protocol of the study was approved by the Paediatrics Review Board and the Regional Ethics Committee of Isfahan University of Medical Sciences (Research Project Number: 394156). Written informed consent was obtained from the parents of selected patients after describing the goal of and treatments in the study.

Their seizures were refractory to all appropriate antiepileptic drugs including valproate, levetiracetam, ethosuximide, topiramate, lamotrigine, clonazepam, clobazam, nitrazepam, zonisamide, and acetazolamide. 

In six cases, ketogenic diet had been tried with no therapeutic effect. These patients had shown no effective response to combination therapy of at least two of these drugs before ACTH administration. 

In this study design, the arbitrary phrase “multiple daily recurrences” was defined as more than ten episodes of seizures per week. Severely intractable epilepsy was defined as multiple daily recurrences and no response to all appropriate anticonvulsants. Severely abnormal EEG was defined as EEGs with more than 80% of tracing being covered with epileptic discharges. 

The patient with multiple daily recurrences and no response to all appropriate anticonvulsants were enrolled into this study.

Inclusion criteria were as follows: Age 1 month-14 yr; severe intractability; severely abnormal EEG; absence of contraindication for ACTH (renal failure, hypertension, cardiac failure, serious infection, peptic ulcer disease, diabetes mellitus...); and Close follow-up. 

Exclusion criteria were severe complication to ACTH: hyperglycemia, hypertension, serious infection; loss of close follow-up; Infantile spasm; and partial seizures with no accompanying generalized seizure. 

Seizure frequency was recorded for a period of 2 wk by patient’s parents before initiation of treatment with ACTH. For all patients, EEG was recorded before treatment.

They received ACTH therapy with synacthen ampule 1mg/1 ml, according to our protocol consisted the dose of 25mcg/kg/day for duration of one week, 12.5 mcg/ kg/day for three days, 12.5 mcg /kg every other day for 4 d, 12.5 mcg/kg every week for duration of 4 wk and 6 mcg/kg every week for duration of 4 wk. Standard treatment with anticonvulsants continued during period of treatment with ACTH for all patients.

All of the cases were followed regularly in Subspecialty Clinic of Pediatric Neurology and evaluated for seizure control and probable side effects. Laboratory tests for probable side effects including Na, K, BS, BUN, Cr, Ca, p, alp, CBC, ESR, CRP was performed 3, 7, 14 d after beginning of treatment. 

EEG finding were recorded before, after termination of ACTH therapy and three months later. All 25 cases had intractable seizure with multiple daily recurrence and severely abnormal EEG before ACTH therapy. 

Clinical features of patients including sex, age at onset of seizure, developmental status, and etiology of epilepsy, MRI findings and prior history of west syndrome are shown in Table 1.

After ACTH, therapy patients were categorized into four groups according to their response to treatment. 

Group 1 with excellent response (more than 80% reduction in seizure frequency), group 2 with moderate response (50%-80% reduction in seizure frequency), group 3 with mild response (20%-50% reduction in seizure frequency) and group 4 with minimal response or no response.

EEG recording was performed in all cases before and after ACTH therapy. 

Short and long-term effects of ACTH therapy for all 25 patients were compared using Freidman test. Wilcoxon signed–rank test was used for comparison of EEG results before and after of ACTH therapy.

## Results

Responses to ACTH therapy were evaluated 3 wk after initiation and 3 months after cessation of ACTH therapy (Table 2). Among 25 patients who had intractable seizure with multiple daily recurrences before ACTH therapy, 18 cases showed more than 50% reduction in seizure frequency 3 wk after initiation of ACTH therapy. However, in 7 of 18 cases (38%), relapse of seizures was observed within 3 months after end of ACTH therapy. Four of eleven cases with excellent response (more than 80% reduction in seizure frequency) and 3 of 7 cases with moderate response (50%-80% reduction in seizure frequency) showed relapse of seizures 3 months after cessation of treatment. In seven cases (38%), no or little (<20%) reduction in seizure frequency was observed 3 months after termination of treatment with ACTH. Reduction in seizure frequency after ACTH therapy was shown in Table 3.

Before treatment, all of the patients had severely abnormal EEG with most of tracing being covered by epileptic discharges. Repeat EEG 3 months after the end of treatment demonstrated more than 50% reduction of epileptic discharge in 16 of 25 (64%) cases.

In this study, there was no relationship between response to ACTH and baseline variables including age, brain MRI, underlying disease and developmental status (P>0.05).

Side effects of ACTH therapy including severe irritability, edema and weight gain with frequencies of 52%, 71%, and 61% were observed respectively (Table 4). Recurrence of seizure was reported before the next dose of ACTH during period of weekly injections in three cases with moderate initial response.

## Discussion

ACTH is considered as one of the most effective available agents in controlling of intractable seizure (5, 7, 14). Various mechanisms have been suggested for antiepileptic action of ACTH; however, the precise mechanism is not fully understood. ACTH acts as a neuropeptide within the brain and it directly may have anti-convulsive properties (14). It also stimulates the synthesis of two allosteric modulators of GABA-A receptors including deoxycorticosterone and tetrahydroxy-corticosterone (15). ACTH has been demonstrated to down-regulate the expression of corticotrophin-releasing hormone (CRH) which results in reduction of pro convulsant activity in the developing brain (16). In addition, ACTH may stimulate glucocorticoid synthesis. Interaction of glucocorticoid with central nervous system steroid receptors is supposed to influence voltage-dependent calcium channels (17).

Totally, 72% of patients achieved favorable response (>50% reduction of seizure frequency) in short-term to ACTH therapy. Follow-up of the patients, 3 months after stopping ACTH therapy, showed recurrence of seizure in 38% of early responders. The excellent effectiveness of ACTH in treatment of infantile spasm but there are only a few studies on effectiveness of ACTH in treatment of patients with intractable seizure other than spasm. 

Snead et al. described their experience of treatment of intractable seizure other than spasms with prednisolone or ACTH. In their study, successful cessation of seizure was achieved in 74% of patients ACTH therapy and in none of the patients treated with prednisolone (5). 

Sinclair used prednisone for treatment of 28 cases with intractable epilepsy. During follow-up period of 1 to 5 yr, 46% of the patients became seizure free and significant decrease in seizure frequency was observed in 40% (18). Steroids were administrated on the treatment of epilepsy beyond infantile spasm in 32 children with intractable seizure. In a study, 25% cases became seizure free, 11% had a seizure reduction of >50% and 11% had a seizure reduction of <50% (7). 

Fifteen children were studied with intractable seizure other than spasm. The seizure was controlled in 13 cases by ACTH therapy but recurrence of seizures was observed in 6 of them within 3 months after stopping ACTH therapy (6). Successful use of ACTH was described in two patients with generalized epilepsy who had refractory to multiple AED (13). 

**Table 1:** Clinical features of patients

**Patient**	**Sex**	**Age at the onset (m)**	**Age (m)**	**Developmental status**	**Etiology**	**Epilepsy syndrome**	**MRI results**	**Comments**
1	F	7	63	Moderate delay	Genetic anomaly	Absence- tonic-GTC	Normal	
2	M	21	78	Severe delay	Encephalitis	Myoclonic-tonic	Diffuse atrophy	
3	M	3	22	Severe delay	Prematurityhypoxic-ischemic encephalopathy	Myoclonic-tonic-GTC	Hypoxic-ischemic change PVL	
4	M	6	36	Severe delay	Unknown	Myoclonic	Normal	History of West syndrome
5	M	30	92	Severe delay	Hypoxic-ischemic encephalopathy	Myoclonic-absence-tonic	Mild Hypoxic-ischemic change PVL-atrophy	
6	M	24	57	Severe delay	Hypoxic-ischemic encephalopathy	Myoclonic-tonic	Hypoxic-ischemic change	
7	M	9	35	Moderate MR	Cerebral dysgenesis	Myoclonic-GTC	Pachygyria	History of West syndrome
8	F	11	67	Severe delay	Unknown	Myoclonic-GTC-tonic	Mild atrophy	
9	M	8	38	Moderate delay	Unknown	Myoclonic-atonic	Normal	History of West syndrome
10	F	7	44	Severe delay	Cerebral dysgenesis	Atonic-tonic-Myoclonic-	Pachygyria	
11	F	3	74	Moderate delay	Hypoxic-ischemic encephalopathy	Myoclonic-GTC	Hypoxic-ischemic change PVL	
12	M	1	28	Severe delay	Syndromic anomaly	Tonic-GTC-Atonic-myoclonic	Normal	
13	F	3	39	Moderate delay	Unkown	Myoclonic-absence-GTC	Mild atrophy	
14	M	1	48	Severe delay	Unknown	Myoclonic-tonic-GTC	Atrophy	
15	F	7	112	Moderate delay	Genetic anomaly	Myoclonic –absence-partial	Normal	
16	M	1	32	Severe delay	Hypoxic-ischemic encephalopathy	Tonic-myoclonic	Hypoxic-ischemic change PVL	
17	M	20	45	Severe delay	Cerebral dysgenesis Microcephaly	Myoclonic	Polymicrogyria	
18	M	8	27	Moderate delay	Hypoxic-ischemic encephalopathy	Partial-GTC	Mild Hypoxic-ischemic change PVL	
19	F	5	94	Moderate delay	Unknown	Myoclonic-tonic-GTC	Mild atrophy	
20	F	1	48	Severe delay	Cerebral dysgenesis	Myoclonic-tonic-absence	Cerebral dysgenesis	
21	M	10	30	Severe delay	Herpes encephalitis	MYOCLONIC-ATONIC-PARTIAL	Bilateral temporal encephalomalacia	Normal development before encephalitis
22	M	8	67	Moderate delay	Cerebral dysgenesis	Myoclonic-GTC-	Pachygyria polymicrogyria	
23	F	5	56	Severe delay	Genetic anomaly	Myoclonic-atonic	Normal	
24	F	9	123	Severe delay	Unknown	Myoclonic-atonic	Normal	
25	F	18	108	Severe delay	Genetic anomaly	Tonic-Atonic—GTC-absence	Normal	

**Table 2:** Results of response to ACTH

**Patients**	**Interracial EEG before ACTH therapy**	**Percent of Reduction of Seizure frequency(3 w after beginning of ACTH)**	**Percent of Reduction of Seizure frequency (3 months after the end of ACTH therapy)**
1	Continuous spike and wave discharge	>80	>80
2	Diffuse Poly spike wave	>80	>80
3	Diffuse Poly spike wave	>80	20-50
4	Diffuse Poly spike wave	>80	>80
5	Diffuse slow spike wave	>80	>80
6	Diffuse slow spike wave	>80	>80
7	Diffuse Poly spike wave	>80	50-80
8	Diffuse slow spike wave	>80	20-50
9	Continuous spike and wave discharge	>80	>80
10	Diffuse Poly spike wave	>80	20-50
11	Diffuse Poly spike wave	>80	>80
12	Diffuse slow spike wave	50-80	50-80
13	Diffuse slow spike wave	50-80	>80
14	Diffuse slow spike wave	50-80	20-50
15	Diffuse slow spike wave	50-80	50-80
16	Diffuse slow spike wave	50-80	<20
17	Diffuse slow spike wave	50-80	50-80
18	Continuous partial spike	50-80	<20
19	Diffuse slow spike wave	20-50	50-80
20	Diffuse slow spike wave	20-50	<20
21	Multifocal spike-polyspike	20-50	<20
22	Diffuse slow spike wave	<20	<20
23	Diffuse Poly spike wave	<20	<20
24	Diffuse slow spike wave	<20	<20
25	Diffuse slow spike wave	<20	<20

**Table 3:** Reduction in seizure frequency after ACTH

**Reduction in seizure frequency (%)**	**3 weeks after** **Beginning of ACTH therapy** **N(%)**	**3 months after** **ACTH therapy** **N(%)**
>80	11 (44)	8 (32)
50-80	7 (28)	5 (20)
20-50	3 (12)	4 (16)
<20	4 (16)	8 (32)

**Table 4:** Side effects of ACTH therapy

**Adverse effects**	**Number of patients**
Edema	18
Weight gain	17
Irritability	15
Increased appetite	13
Limpness	7
Lethargy	5
Anorexia	4
Abdomen abnormality(distension)	4
Vomiting	3
Recurrence of seizures before next dose	3
Thrush	2
Soft tissue infection	2
Hyperglycemia	1
Hypokalemia	1
Hypertension	0

Steroids and particularly ACTH may be useful in treatment of children with intractable seizure but most of them indicate that effects of steroids were transient in half of the patients with initial significant response. 

The long-term efficacy was reported of ACTH therapy in controlling seizure in two patients. Long-term weekly ACTH therapy for duration of one year was effective and seizure was completely controlled (12). 

In our study, recurrence rate of seizure was less than 50%. This difference may be ascribed to several factors such as drug dose, interval of ACTH injection, duration of treatment, type of seizure, number of patients, different etiology.

Several factors may be important in effectiveness of ACTH in intractable seizures. Underlying disease, abnormalities of brain imaging, seizure types, duration of treatment, dose and interval of ACTH injection probably are the most important determinants, which may play a role in effectiveness of treatment.

In our study, excellent response was observed in patients with history of infantile spasm. More than 80% reduction in seizure frequency in two and 50%-80% reduction in seizure frequency in one of these three patients with history of infantile spasm was observed 3 months after cessation of treatment with ACTH. 

Although the number is too little for a definitive conclusion but this finding may suggest better response to ACTH in patients with history of infantile spasm. 

Most of our cases had polymorph seizure (21 out of 25). Video monitoring has not been performed for most of our cases, therefore, we could not exactly determine all seizure types and effects of ACTH on different types of seizure but according to parental reports, myoclonic and absence seizures were more responsive to ACTH.

In this study, there was no relationship between response to ACTH and baseline variables including age, brain MRI, underlying disease and developmental status but because of small sample size importance of this finding could not be overvalued.

We observed frequent side effects including irritability, edema and weight gain in most of our patients but in no cases, a serious problem was observed. In cases with probable side effects, complete clinical and laboratory evaluation was performed and after rule out of serious side effects, treatment was continued. Similar side effects have been reported in previous studies in which ACTH has been used (4, 8).

In our study, in no patient, seizure frequency increased, but recurrence of seizures, though with less frequency, was observed in three of the cases with moderate initial response before next dose of weekly ACTH injections.

For treatment purpose in these cases, injection interval decreased to 5 d with good result.

Because of high frequency of mentioned side effects, which causes severe concern and anxiety in patient’s family and may finally end to premature cessation of treatment, we recommend that hospitalization of all patients for a period of 10-14 d at the onset of treatment and a close follow up of the patients be considered during treatment period.

In our study, we have not performed video EEG monitoring in most of our patients so we could not exactly determine all seizure types in the patients. In addition, we did not hospitalize the patients and follow the response to video monitoring. Because of this inevitable limitation, response to ACTH was judged according to parent’s recorded observations. 

There is no clear data regarding appropriate dose, duration, and interval of drug administration. This drug may be more effective for treatment of pharmaco resistant seizures if proper dose, interval, and duration of treatment could be determined. Further large-scale studies focusing on these limitations are required to clarify these important issues.

**In Conclusion, **ACTH therapy is relatively safe and effective for patients with intractable seizures. Our result is consistent with the few studies reporting the effective ACTH treatment in children with intractable epilepsy other than spasm. 
